# The efficacy and safety of multiple sessions of multisite transcranial random noise stimulation in treating chronic tinnitus^☆^

**DOI:** 10.1016/j.bjorl.2018.05.010

**Published:** 2018-06-28

**Authors:** Samer Mohsen, Akram Pourbakht, Mohammad Farhadi, Saeid Mahmoudian

**Affiliations:** a International Campus of Iran University of Medical Sciences, School of Rehabilitation Sciences, Department of Audiology, Tehran, Iran; b Damascus University, School of Medicine, Department of Otolaryngology, Damascus, Syria; c Iran University of Medical Sciences, School of Rehabilitation Sciences, Department of Audiology, Tehran, Iran; d Iran University of Medical Sciences (IUMS), ENT and Head & Neck Research Center, Tehran, Iran

**Keywords:** Tinnitus, Electrical stimulation, Transcranial random noise stimulation, Multiple-sessions, Safety, Zumbido, Estimulação elétrica, Estimulação transcraniana por ruído aleatório, Múltiplas sessões, Segurança

## Abstract

**Introduction:**

Random noise stimulation was reported as the more effective and safer type of electrical stimulation techniques in relieving tinnitus symptoms. The multisite protocol of transcranial random noise stimulation has shown additional favorable effects.

**Objective:**

Here we will discuss the role of applying eight sessions of multisite transcranial random noise stimulation in decreasing tinnitus loudness and annoyance without exerting additional adverse effects.

**Methods:**

Twenty-nine subjects (8 female), the mean age of (45.34 ± 9.57) with chronic tinnitus received transcranial random noise stimulation in the multisite protocol, 10 min of auditory-transcranial random noise stimulation applied over the T3, T4 preceded by 10 min of prefrontal-transcranial random noise stimulation applied over F4, FP1. In the first group, only one session was applied and the multiple-sessions group contained eight repeated sessions. Visual analog scale scores for loudness and distress were recorded before and immediately after the treatment. Multivariate repeated measure ANOVA test was used and minimal detectable change calculated.

**Results:**

There was a statistically and clinically significant reduction in tinnitus loudness and annoyance in both groups (*p* < 0.05, effect size (*η*^2^) > 0.8), while the amount of annoyance suppression in the multiple-sessions group was significantly greater than the single-session group. The patients of the multiple-sessions transcranial random noise stimulation group reported an improvement in their sleep and lower tinnitus handicap inventory scores without experiencing any additional adverse effects of the intervention.

**Conclusions:**

The results of this study showed a substantial improvement in tinnitus symptoms by using the multiple sessions of transcranial random noise stimulation in the multisite protocol without producing any additional side effects. We suggest further clinical trials with long-term follow-up be investigated.

## Resumo

**Introdução:**

Tem sido relatado que a estimulação transcraniana por ruído aleatório é um tipo mais eficaz e seguro de técnica de estimulação elétrica no alívio dos sintomas do zumbido. O protocolo multissítio da estimulação transcraniana por ruído aleatório mostrou efeitos favoráveis adicionais.

**Objetivo:**

Discutir o papel da aplicação de oito sessões de estimulação transcraniana por ruído aleatório multissítio na diminuição da intensidade (*loudness*) do som e incômodo (*annoyance*) do zumbido sem causar efeitos adversos adicionais.

**Método:**

Vinte e nove indivíduos com zumbido crônico (oito do sexo feminino), com média de 45,34 ± 9,57 anos, receberam estimulação transcraniana por ruído aleatório no protocolo multissítio, 10 minutos de estimulação transcraniana por ruído aleatório-auditivo aplicado no T3, T4 precedido por 10 min de estimulação transcraniana por ruído aleatório pré-frontal aplicado sobre F4, FP1. No primeiro grupo, apenas uma sessão foi aplicada e o grupo de múltiplas sessões recebeu oito sessões. Os escores da escala visual analógica para a intensidade do som (*loudness*) e incômodo *(annoyance*) do zumbido foram registrados antes e imediatamente após o tratamento. O teste Anova multivariada para medidas repetidas foi utilizado e a mudança mínima detectável foi calculada.

**Resultados:**

Houve uma redução estatisticamente e clinicamente significante na intensidade e incômodo do zumbido em ambos os grupos (*p* < 0,05; tamanho do efeito (η2) > 0,8), embora o nível de supressão do incômodo no grupo de sessões múltiplas tenha sido significantemente maior do que no grupo de sessão única. Os pacientes do grupo estimulação transcraniana por ruído aleatório com múltiplas sessões relataram uma melhoria no sono e menores escores no *tinnitus handicap inventory*, sem experimentar quaisquer efeitos adversos adicionais da intervenção.

**Conclusão:**

Os resultados deste estudo mostraram uma melhora substancial nos sintomas do zumbido utilizando múltiplas sessões de estimulação transcraniana por ruído aleatório no protocolo multissítio sem produzir quaisquer efeitos colaterais adicionais. Sugerimos que novos ensaios clínicos com seguimento de longo prazo sejam realizados.

## Introduction

Millions of people around the world are suffering from tinnitus. They have the auditory phantom sound in their ears or head.^1^ Between 6% and 25% of them have debilitating symptoms^2^ and seek medical services for problems with their daily activity, sleep, concentration and emotional functions.^3^ Apart from some reversible acute conditions, tinnitus remains one of the most challengeable situations at the otology clinics. Several treatment approaches were introduced for the management of tinnitus. Among these techniques, we name transcranial Electrical Stimulation (tES) which was frequently used in tinnitus research in the Direct Current (DC) form^4^ and more recently in the form of transcranial Random Noise Stimulation (tRNS) which have got more popular recently for its superiority in relieving tinnitus symptoms with fewer side effects than other models of Tes.^5^, ^6^ This technique was introduced by Terney et al., 2008 as a novel model of tES, for more details about the tRNS paradigm we refer to their paper.^7^

Tinnitus is defined as an auditory disorder,^1^ however, in chronic cases, the involvement of the central nervous system is inevitable.^8^, ^9^ That includes the auditory cortex as the core system accompanied by attention,^10^, ^11^ memory,^12^, ^13^, ^14^ and limbic systems.^15^, ^16^ The brain areas involving these systems share some mal-adaptive features which have been recently recognized by means of neuroimaging techniques or functional connectivity analysis, which led to the concept of tinnitus network.^17^, ^18^ Moreover, the longer the period of tinnitus is, the more complicated scene of these abnormal connections might be seen.^19^, ^20^ Such model can interpret the wide range of emotional, functional and catastrophic symptoms the patients report, and also give a new insight for the management approach like neuromodulation. tRNS can modulate the cortical activity by introducing low-intensity electrical currents to the cortex.^21^ There are encouraging data concerning tRNS effects on tinnitus symptoms,^5^, ^20^ anxiety and depression disorders.^22^ The targeted areas were the prefrontal and auditory cortices, and recently the both of them in multisite protocols.^23^ It was reported that prefrontal tRNS can modulate the activity of distress network composed of the prefrontal cortex, the anterior cingulate cortex, amygdala, and parahippocampus (personal communication). So, considering tinnitus chronicity and mal-plasticity we can hypothesize that multiple sessions of neuromodulation are needed to produce effective clinical improvement, albeit the results of single sessions were reasonably good but not enough to relieve the patients suffering.

Neuromodulation techniques can improve tinnitus symptoms by decreasing its loudness and annoyance; however, there is no evidence that they can cure tinnitus. In medicine, when a therapeutic approach is used for managing a disorder or palliating its symptoms, it needs to be cost-effective and safe in order to be approved for the clinical use. There are strong data about the cost-effectiveness and safety of tRNS.^24^, ^25^ However, by introducing the multisite protocol for tinnitus we aimed in this study to investigate whether the multiple sessions of this protocol can yield more desirable effects without producing any more adverse effects.

## Methods

### Participants

Twenty-nine subjects (8 female), the mean age of (45.34 ± 9.57) with chronic non-pulsatile tinnitus (duration > 6 months) participated in this study. The participants did not receive any treatments for their tinnitus or any therapeutic interventions on brain or ears (acoustical, electrical, etc.) during the 3 months before the experiment starting day. Participants with neurological disorders such as head trauma, brain tumors, as well, those who were receiving medications for mental or psychological disorders were excluded. Any other cases which may interfere with using tRNS were excluded like epilepsy, severe organic co-morbidity, and having pacemaker or defibrillator or current pregnancy. All participants filled in the items of the Persian version of tinnitus handicap inventory (THI-P),^26^ which contains 25 items with three forced choices (Yes, No and Sometimes) and a total score range of 0–100.^27^ Scores between 38 and 76, which refer to moderate to severe tinnitus were accepted for recruitment. In order to get a more homogenous group, all patients were given the validated Persian version of Hospital Anxiety and Depression Scale (HADS)^28^ and those with a score of 21 or less (less than 11 for either depression and anxiety subscales, referring not to have depression or anxiety disorders), were included.^29^ The participants were referred to our ENT clinic to have a complete examination in order to exclude all treatable cases of tinnitus.

### Tinnitus psychoacoustic assessments

First, for all participants, the Pure-Tone Thresholds were measured and then, psychoacoustic assessments including Pitch Matching of Tinnitus (PMT) and Loudness Matching of Tinnitus (LMT) were performed in a soundproof chamber. Pitch- and loudness-match tests were performed contralateral to the tinnitus ear for unilateral cases with tinnitus, or contralateral to the ear with most bothersome tinnitus in cases of bilateral tinnitus. Participants were asked to identify which pitch best matched the Pitch of Their Tinnitus (PMT) and to identify the level of the external sound to be equivalent to the Loudness of their Tinnitus (LMT).

### Transcranial random noise stimulation (tRNS)

tRNS was delivered using a portable battery-driven constant current stimulator device. The intensity of the currents was set at 2 mA without DC cutoff. Two frequency ranges were used, low-frequency tRNS (lf-tRNS: 0.1–100 Hz) for the auditory cortex stimulation and high-frequency tRNS (hf-tRNS: 100–640 Hz) for the prefrontal stimulation. The current was transmitted using two surface electrodes coated with a pair of saline-soaked sponges 35 cm^2^ in size. The total period of treatment session was fixed at 20 min. Each participant received 10 min of DLPFC-tRNS followed by 10 min of AC-tRNS. The alternating current was increased in a ramp-like mode to reach the desired intensity with a rise and fall time of 30 s.

### Experimental design

In this clinical trial, the participants were randomly allocated into two groups, the single-session stimulation group (17 participants) and the multiple-sessions group (12 participants). The two groups were matched for age, gender, tinnitus duration, and type, THI and HADS scores see Table 1. Multiple sessions were given twice per week with a total of 8 sessions accomplished during 4 weeks. The subjective evaluation was performed before and immediately after the treatment. This study was a part of a registered randomized clinical trial and all procedures performed in this study were in accordance with Ethics Committee of Iran University of Medical Sciences (1395.9321667001, 22/4/2017), and with the 1964 Helsinki declaration and its later amendments or comparable ethical standards. After getting all the information regarding the research methodology, all patients had given a written informed consent for their participation. Before participation, all qualified patients were invited to voluntarily participate in our research and after completing their participation, patients who declared a need for getting more help were referred to the tinnitus Clinic, to benefit from the treatment program available in our center.

**Table 1:** Tinnitus characteristics for the two groups separately and the whole sample.

	Single-session tRNS	Multiple-sessions tRNS	*p*-values
Gender (M/F)	12/5	9/3	0.56
Age (mean)	48.42 ± 9.56	43.18 ± 9.26	0.15
Tinnitus duration (month)	48.53 ± 47.04	66.13 ± 52.68	0.22
Type (PT/NBN)	12/5	10/2	0.28
Laterality (uni/bil)	10/7	8/4	0.31
PMT (mean)	5.65 ± 1.27	5.17 ± 1.03	0.29
LMT (mean)	6.71 ± 3.25	5.92 ± 2.31	0.45
THI (mean)	52.53 ± 13.45	52.83 ± 13.33	0.92
HADS depression (mean)	7.05 ± 2.01	6.5 ± 1.16	0.39
HADS anxiety (mean)	6.49 ± 2.32	6.58 ± 1.37	0.63

M, Male; F, Female; PT, Pure Tone; NBN, Narrow Band Noise; uni, unilateral; bil, bilateral.

### Evaluation

The Visual Analog Scale (VAS) was used to assess the tinnitus Loudness (VAS-L) and Annoyance (VAS-A). Each participant was asked to give a degree from 0 to 10 depending on the intensity of his problem. For example, the examiner asking “How loud is your tinnitus? and the patient answer is ranging from 0 to 10; 0 = no tinnitus and 10 = as loud as intolerable”, and (“How annoying is your tinnitus; 0 = No Annoyance and 10 = Extreme Annoyance”). For the multiple-sessions group, THI score also was measured after the end of the multiple-sessions treatment.

For side effects, we used a questionnaire-like sheet for identifying the presence and severity of any side effects like headache, fatigue, tingling, itching, burning or a pain sensation, nervousness and difficulties in concentrating, or any other unpleasant sensation such as drowsiness or nausea.^30^ The participant had to answer these questions after each session of tRNS, therefore for the multiple-sessions group, we considered the side effects for all sessions. The presence of the side effects was coded in a binary system (no = 0, yes = 1) and the severity was rated using a Numerical Analog Scale (NAS) from one to five, one being very mild and five being extremely intense.^30^

### Statistical analysis

Statistical analysis was performed using the SPSS version 19.0 software package (SPSS Inc., Chicago, IL). The Kolmogorov–Smirnov test was used to investigate the normal distribution of data which had a normal distribution (*p* > 0.05). The statistical significance was tested using the Global Linear models in SPSS toolbox for repeated measures and the pairwise comparison to find the differences used Bonferroni. Furthermore, an independent *t*-test was conducted for comparing the amount of suppression (the score difference between pre- and post-stimulation) between the single session and multiple sessions of tRNS for tinnitus loudness and annoyance. Also, a dependent *t*-test was used for comparing the THI score differences before and after stimulation in the multiple-sessions group. In addition to statistical significance, the clinical significance was investigated by means of the Minimal Detectable Change (MDC%). The MDC, at 95% Confidence, was calculated from the Standard Error of Measurement (SEM) to indicate a real change for individual patients.^31^, ^32^

The statistical analysis of presence and severity of side effects between the two groups were performed using Mann–Whitney test.

## Results

### tRNS outcome measures

**Figure 1:**
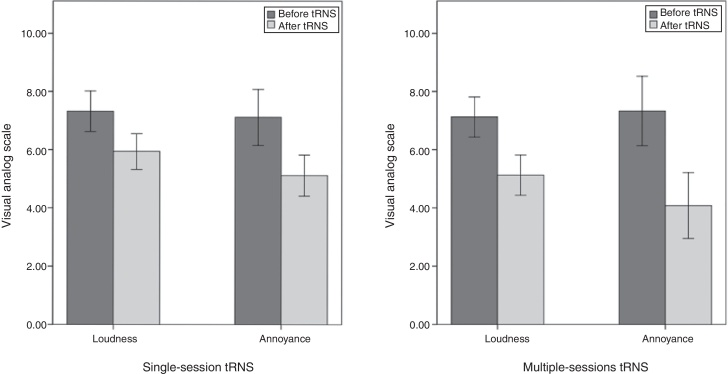
Overview of the obtained results for tinnitus loudness and annoyance after a single session or multiple sessions of random noise stimulation (tRNS). *Y*-axis is the VAS scores mean.

A repeated measure ANOVA was conducted for the Visual Analog Scale (VAS-L) score as a within-subjects variable with two levels (before and after stimulation) and the group (single and multiple-sessions tRNS) as a between-subjects variable. The results revealed that there was a statistically significant main effect for the VAS-L measurement (*F* [1,27] = 191.53, *p* < 0.001, *η*^2^ = 0.87). As well as, a statistically significant interaction effect was noticed for the VAS-L and the group variables (*F* [1,27] = 6.38, *p* = 0.018, *η*^2^ = 0.19). Simple main effects analysis using Bonferroni showed a significant (*p* < 0.001) reduction in tinnitus loudness after tRNS for both groups, see Table 2 for loudness scores means before and after tRNS as well as the MDC% values and the percentage of mean difference. Another resembling ANOVA test was conducted for the tinnitus annoyance measured by VAS-A score; There was a statistically significant main effect for the VAS-A (*F* [1,27] = 125.38, *p* < 0.001, *η*^2^ = 0.82) and also a statistically significant interaction between the VAS-A, and the group variables (*F* [1,27] = 7.11, *p* = 0.013, *η*^2^ = 0.21). Simple main effects analysis revealed that there was a significant (*p* < 0.001) reduction in tinnitus annoyance after tRNS in both groups (Table 2) (Fig. 1).

**Table 2:** Mean differences of visual analog scale & THI scores before and after tRNS.

	Factor name	Before treatment M ± SD	After treatment M ± SD	MD%	MDC%
Single-session tRNS	VAS-L	7.32±0.3	5.94±0.27	19.09	11.72
	VAS-A	7.12±0.45	5.12±0.37	26.93	16.52

Multiple-session tRNS	VAS-L	7.13 ± 0.36	5.12 ± 0.33	28.24	10.60
	VAS-A	7.33 ± 0.54	4.08 ± 0.44	45.04	17.28
	THI	52.83 ± 13.33	36.17 ± 20.04	36.19	22.33

MD%, percentage of Mean Differences; MDC%, percentage of Minimal Detectable Change.

### THI scores comparison in the multiple-sessions group

Since there was no long-term follow-up in this study, we considered the THI differences measure only in the multiple-sessions group. There was a significant reduction in THI scores after the four weeks of tRNS in comparison to before it (*t* [11] = 6.66, *p* < 0.001), (Table 2). Most participants had reported an improvement in their sleep during the four weeks of treatment.

### Single versus multiple sessions

**Figure 2:**
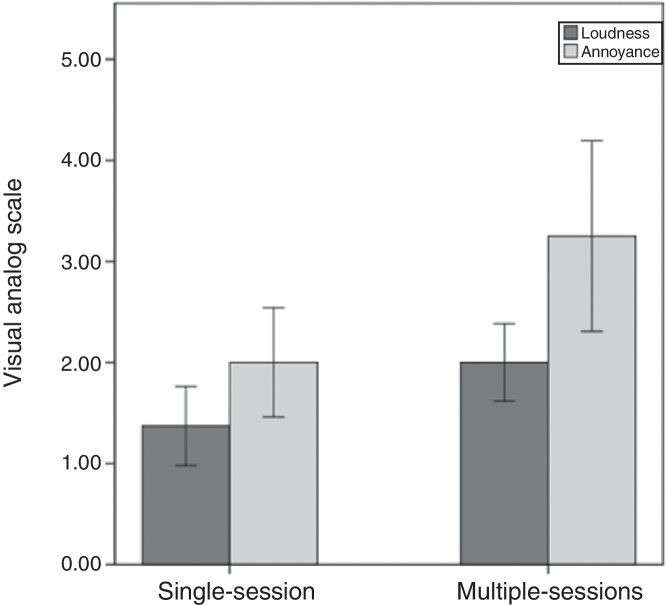
The amount of suppression for tinnitus loudness and annoyance in both single-session and multiple-sessions tRNS groups. *Y*-axis is the VAS scores difference mean.

Comparing the amount of suppression between the single and multiple sessions of tRNS showed a significant increased suppression for multiple-sessions (M = 2, SD = 0.6) in comparison to the single-session (M = 1.37, SD = 0.7) for tinnitus loudness (*t* [27] = −2.38, *p* = 0.02). Another significant increased suppression effects was also obtained for tinnitus annoyance (*t* [27] = −2.66, *p* = 0.01) in the multiple-sessions group (M = 3.25, SD = 1.4) in comparison to the single-session (M = 2, SD = 1.04) (Fig. 2).

### Side effects measures

Table 3 shows the presence of side effects in each group and the severity of each reported side effect. There were no significant differences in both occurrence and severity of side effects reported between groups (*p* > 0.05).

**Table 3:** Adverse effects of tRNS after stimulation in the single and multiple-sessions group.

	Headache		Tingling		Nervousness		Pain		Fatigue	Itching	Burning	Drowsiness	Nausea	Total
	*N*	Mean intensity	*N*	Mean intensity	*N*	Mean intensity	*N*	Mean intensity	*N*	*N*	*N*	*N*	*N*	*N*
Single-session	1	1 ± 0	7	1.2 ± 0.4	0	–	1	1 ± 0	0	0	0	0	0	17
Multiple-sessions	1	1 ± 0	6	1.33 ± 0.5	1	2 ± 0	0	–	0	0	0	0	0	12

*N*, frequency of occurrence. Mean intensity for reported side effects only (a score from 5).

## Discussion

The results of this study revealed a substantial improvement in tinnitus loudness and annoyance after tRNS treatment in both groups. However, the amount of suppression was remarkably larger in the multiple-sessions tRNS group accompanied by an important decrease of THI scores. Auditory cortex tRNS was reported to be more effective in reducing tinnitus loudness and annoyance than tDCS and also Tacs,^5^ therefore, on the basis of tRNS superiority revealed by this study, we conducted our new research in which we aimed to find out the efficacy of using multiple sessions of multisite tRNS in comparison to a single session outcomes. The results of our study are in coherence with the data reported for multiple sessions using other models of neuromodulation.^33^, ^34^ Moreover, these results had a clinical importance which means that the reported statistically significant effects refer to actual improvements of patients symptoms supported by a large effect size and a reasonable mean difference in comparison to the minimal detectable change (Table 2). An earlier study showed that the multiple sessions of auditory tRNS had resulted in more suppression of tinnitus loudness but not annoyance,^25^ the use of multiple session of our multisite protocol yielded more suppression over both loudness and annoyance, however the mean difference percentage (MD%) of annoyance suppression in the multiple-sessions group was remarkably greater than loudness MD% as seen in Table 2. The proposed mechanism of such findings based on the role of modulating the distress network through modulating the altered function of the dorsolateral prefrontal cortex.^35^, ^36^, ^37^ Some cautions should be taken here because we did not use the sham stimulation as a placebo in this study, albeit, it was used in our previous study for single session tRNS and the result showed no placebo effects of tRNS.^38^

On the other hand, THI is the most clinically used questionnaire for tinnitus, in which we can explore the impact of tinnitus on the patient's functions and daily life.^39^ Improving the THI scores by means of multiple sessions of tRNS refers to an improvement of patient daily activities. Such effects can be interpreted by a plasticity reorganization likely occurred and promoted repeatedly after each session of tRNS which might lead to modulation of the abnormal activity in the overlapping distress and salience networks involved in tinnitus handicap. More researches are needed to prove this hypothesis. The most important aspect our patients reported is improving their sleep which is a noteworthy finding and can be interpreted both by decreasing tinnitus loudness which disturbs the patient before sleep, or more often by decreasing tinnitus annoyance, the main factor that causes difficulty in falling asleep,^40^ moreover, the modulation of limbic system function is a probable proposed factor.

Our study results highlighted the safety aspect of multiple sessions of tRNS. In fact, the nature of the RN current, which is a type of alternative current, in which the direction of flow varies continuously and the cathode-anode electrodes replace each other in every half cycle,^41^ is an important factor in lessening the adverse effects seen under the anode electrode of Direct Current Stimulation (tDCS) but not by Trns.^42^ Moreover, the perceived sensations of tRNS are substantially less intense than tDCS resulting in a lower level of discomfort reported by participants.^43^ Adding to all, the proper use of this technique (stimulation parameters and set up) is another golden key. Therefore, in our experiment the reported adverse effects were negligible.

## Conclusion

The results of this study showed a substantial improvement in tinnitus symptoms by using the multisite protocol of tRNS in a single session and multiple sessions. The results of multiple sessions of tRNS were more remarkable, and the amount of suppression difference between the two groups was substantial for tinnitus-related annoyance measured by VAS-A. The fact that the multiple sessions did not produce any additional side effects is another compelling result. Therefore, we recommend using the multiple sessions of the multisite protocol of tRNS for chronic tinnitus with annoyance, albeit, we should do such recommendation with some cautions because of the small sample size of our research subgroups. However, we did not report the long-term effects of our protocol because of some limitations that constricted our study design; so, a further long-term sham-controlled clinical trial with a larger sample size is suggested.

## Funding

This study was a part of a randomized clinical trial registered at Iranian Registry of Clinical Trials (Identifier: 20586 30/6/2017).

## Conflicts of interest

The authors declare no conflicts of interest.
